# The impact of bicuspid aortic valve morphology on von Willebrand factor function in patients with severe aortic stenosis and its change after TAVI

**DOI:** 10.1007/s00392-022-02047-6

**Published:** 2022-07-15

**Authors:** Nastasia Roth, Carolin Heidel, Congde Xu, Ute Hubauer, Stefan Wallner, Christine Meindl, Andreas Holzamer, Michael Hilker, Marcus Creutzenberg, Samuel Sossalla, Lars Maier, Carsten Jungbauer, Kurt Debl

**Affiliations:** 1 Department of Internal Medicine II, University Medical Center, Regensburg, Germany; 2 Department of Clinical Chemistry and Laboratory Medicine, University Medical Center, Regensburg, Germany; 3 Department of Cardiothoracic Surgery, University Medical Center, Regensburg, Germany; 4 Department of Anesthesiology, University Medical Center, Regensburg, Germany

**Keywords:** von Willebrand factor, Acquired von Willebrand syndrome, Bicuspid aortic valve, Aortic stenosis, Transcatheter aortic valve implantation

## Abstract

**Background:**

Aortic stenosis (AS) can cause acquired von Willebrand syndrome (AVWS) and valve replacement has been shown to lead to von Willebrand factor (vWF) recovery. The aim of the current study was to investigate the prevalence of AVWS in different severe AS phenotypes and its course after transcatheter aortic valve implantation (TAVI).

**Methods:**

143 patients with severe AS undergoing TAVI were included in the study. vWF function was assessed at baseline, 6 and 24 h after TAVI. AVWS was defined as a reduced vWF:Ac/Ag ratio ≤ 0.7. Phenotypes were classified by tricuspid (TAV) and bicuspid (BAV) valve morphology, mean transvalvular gradient (*P*_mean_), stroke volume index (SVI), ejection fraction (EF) and indexed effective orifice area (iEOA).

**Results:**

AVWS was present in 36 (25.2%) patients before TAVI. vWF:Ac/Ag ratio was significantly lower in high gradient compared to low-gradient severe AS [0.78 (IQR 0.67–0.86) vs. 0.83 (IQR 0.74–0.93), *p* < 0.05] and in patients with BAV compared to TAV [0.70 (IQR 0.63–0.78) vs. 0.81 (IQR 0.71–0.89), *p* < 0.05]. Normalization of vWF:Ac/Ag ratio was achieved in 61% patients 24 h after TAVI. As in the overall study cohort, vWF:Ac/Ag ratio increased significantly in all severe AS subgroups 6 h after TAVI (each *p* < 0.05). Regarding binary logistic regression analysis, BAV was the only significant predictor for AVWS.

**Conclusions:**

BAV morphology is a strong predictor for AVWS in severe AS. TAVI restores vWF function in most patients with severe AS independently of AS phenotype and valve morphology.

**Graphical abstract:**

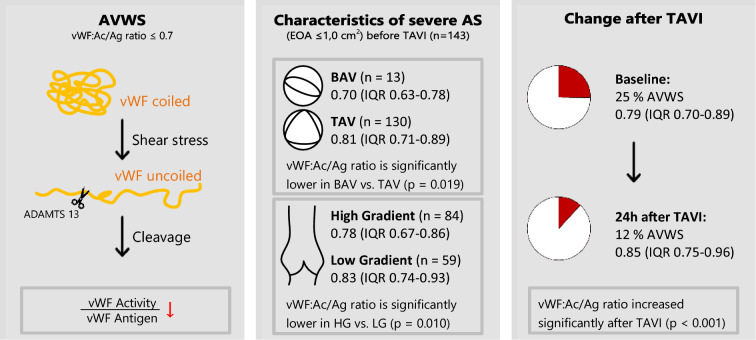

## Introduction

Heart valve disease can be associated with acquired type 2a von Willebrand syndrome (AVWS), characterized by a selective deficiency of high-molecular-weight von Willebrand multimers (HMWM) [1]. The shear stress caused by valvular stenosis or regurgitation induces the cleavage of HMWM by the protease ADAMTS13 resulting in bleeding tendency [1, 2]. Heyde’s syndrome, first described in 1958, refers to the association between aortic valve stenosis (AS) and bleeding from angiodysplasia [3, 4]. AVWS has been evaluated in several cardiovascular diseases [5]. Previous studies have shown that mean transvalvular gradient (*P*_mean_) correlates with vWF function loss in patients with severe AS [6, 7]. Interestingly, AVWS may resolve after surgical and transcatheter aortic valve implantation (TAVI) [8, 9]. Furthermore, monitoring of HMWM defects during TAVI is predictive of the presence of aortic regurgitation and has prognostic implications [10]. Current guidelines propose an AS classification system by mean transvalvular gradient, ejection fraction (EF) and stroke volume index (SVI) [11]. However, vWF function has not been investigated in these AS subgroups. Additionally, influence of bicuspid (BAV) and tricuspid (TAV) valve morphology on vWF function has not been studied yet.

The aim of this study was therefore to investigate the prevalence of AVWS in different subgroups and valve morphologies of severe AS and its course after TAVI.

## Methods

### Study population

**Fig.1:**
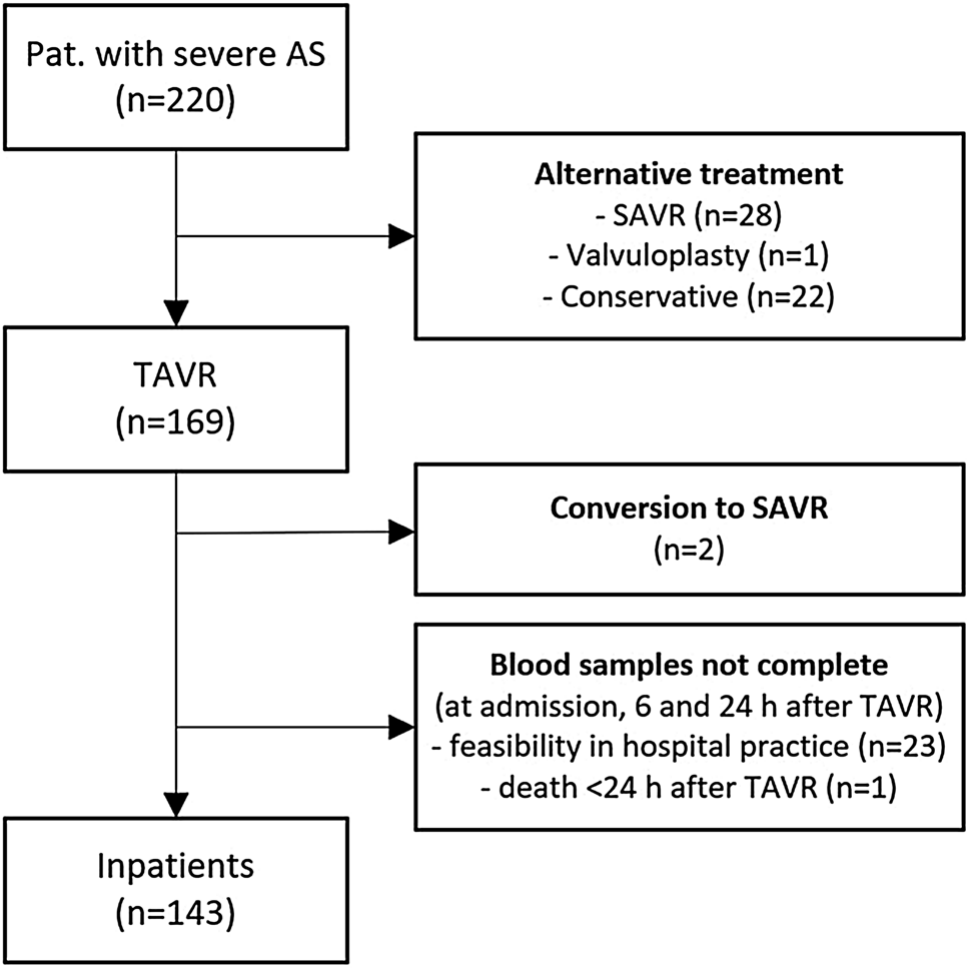
Flowchart patients

From May 2017 to October 2018, 143 patients with severe symptomatic AS presenting at the University Medical Center Regensburg were prospectively enrolled in the investigator initiated single-center study. All included patients gave informed consent, and the study was approved by the local ethics committee. No external funding was obtained to support the study. Clinical data were collected from all participants. The decision for TAVI was made by the local Heart Team after careful evaluation of each case (Fig. 1).

### Classification of severe AS phenotypes and valve morphology

AS severity and phenotype were assessed by transthoracic echocardiography (TTE) using an integrated and stepwise approach. To define the different phenotypes of severe AS (valve area ≤ 1.0 cm^2^) the new classification system by gradient, flow and ejection fraction was used [12]: high gradient (*P*_mean_ ≥ 40 mmHg) vs. low gradient (*P*_mean_ < 40 mmHg); normal flow (SVI ≥ 35 ml/m^2^) vs. low flow (SVI < 35 ml/m^2^), preserved ejection fraction (EF ≥ 50%) vs. reduced ejection fraction (EF < 50%). The influence of indexed effective orifice area (iEOA) was analyzed comparing iEOA < median vs. iEOA ≥ median. Anatomic evaluation and classification of TAV and BAV were based on established criteria using echocardiography and cardiac computed tomography [13].

### Procedural data and device success

TAVI was performed in a hybrid operating room using balloon-expandable (48.3%) or self-expandable (51.7%) aortic valve protheses of the second generation. Transfemoral access with two ProGlides (Abbott Vascular, Abbott Park, Illinois, USA) or a MANTA vascular closure device (Teleflex, Wayne, Pennsylvania) was used in 132 patients (92.3%). Transapical (6.3%) and transsubclavian approaches (1.4%) were used if transfemoral access was not feasible. Vascular complications and bleeding events were carefully assessed according to VARC-2 criteria [14]. Prosthetic valve function was evaluated by TTE before discharge using established criteria [14, 15]. Paravalvular leak (PVL) was referred to as significant if it was at least moderate (≥ II°) corresponding to VARC-2 criteria. The measurements of mean transprosthetic gradient (*P*mean after TAVI) were not conducted in three patients, iEOA after TAVI was not measured in 11 patients. Severe patient–prosthesis mismatch (PPM) was defined as iEOA < 0.65 cm^2^/m^2^ for BMI < 30 kg/m^2^ and iEOA < 0.60 cm^2^/m^2^ for BMI ≥ 30 kg/m.^2^. [14]

### Blood sampling and laboratory analysis

vWF activity (vWF:Ac), vWF antigen (vWF:Ag) and activity-to-antigen-ratio (vWF:Ac/Ag ratio) were assessed at baseline before TAVI and 6 h and 24 h after TAVI. vWF:Ac was analyzed using the Innovance^®^ vWF Ac system provided by SIEMENS (Siemens Healthcare GmbH, Erlangen/Germany), and vWF:Ag was measured by immunoturbidimetry using the measuring devices of Siemens Healthcare Diagnostics or Sysmex (Sysmex Corporation). AVWS was defined as vWF:Ac/Ag ratio ≤ 0.7 [16]. vWF:Ag and vWF:Ac levels above measurable range were determined as the highest measurable value, each at 600 U/dl.

### Statistics

In descriptive statistics for not normally distributed data median with interquartile range (IQR) was used. To assess correlations between two variables Spearman’s rank correlation coefficient was calculated. Non-normally distributed, independent variables were tested with Mann–Whitney-*U* test. To compare categorical variables the Chi-square test was used. To evaluate related variables Wilcoxon signed rank test was used. Among different parameters characterizing AS, predictors for AVWS were identified with binary logistic regression analysis with backward elimination. Commercially available statistical software was used for analysis (IBM SPSS statistics 25, SPSS Inc., Chicago, Illinois).

## Results

### Study population and baseline vWF parameters

**Fig.2:**
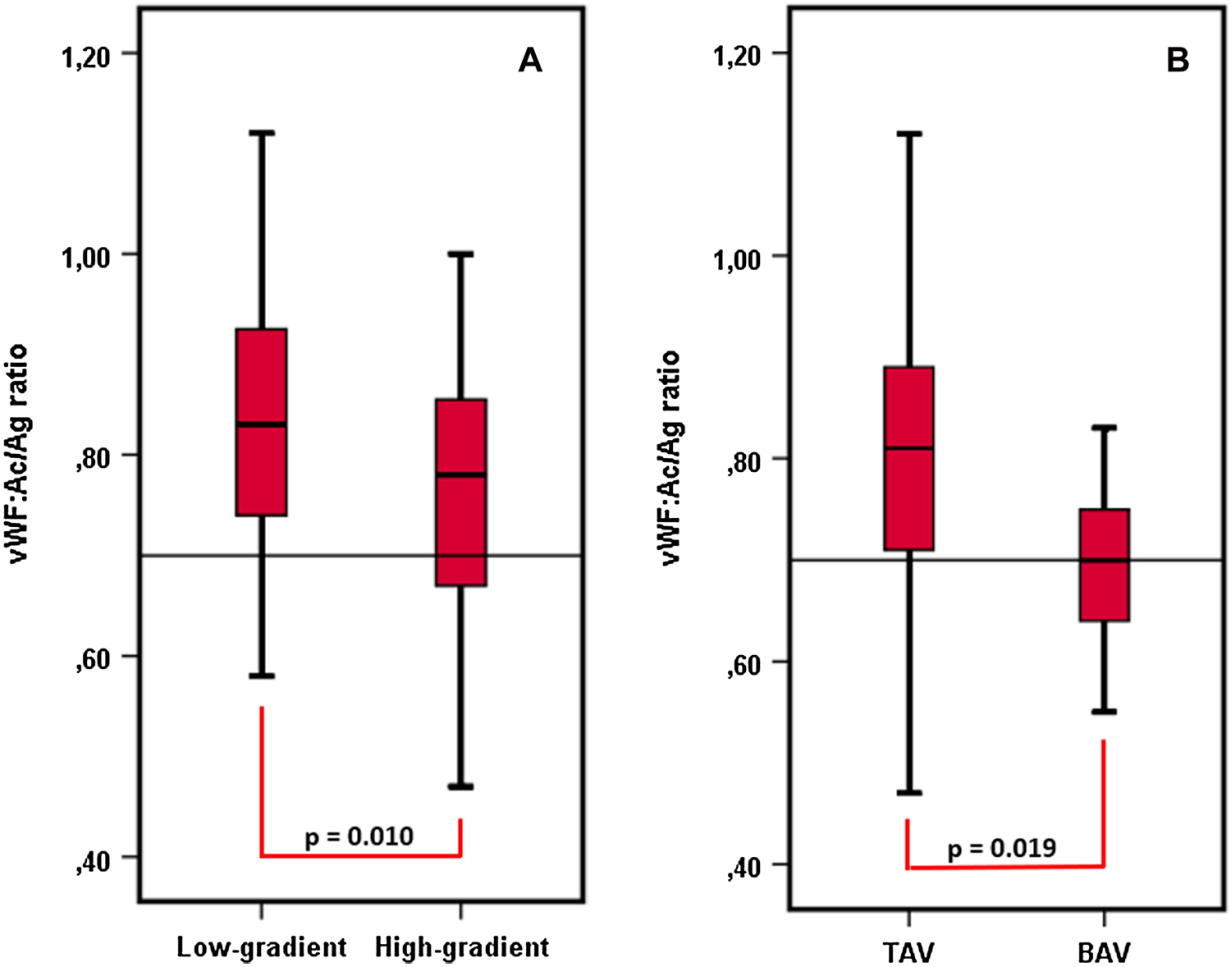
vWF function at baseline—mean gradient and valve morphology. Boxplots: vWF:Ac/Ag ratio at baseline depending on valve morphology and mean transvalvular gradient (low gradient compared to high gradient at baseline *p* = 0.010; TAV compared to BAV, *p* = 0.019)

Baseline characteristics of the study population are depicted in Table 1. The median patient age was 82 years (IQR 78–85) and 55.2% were men. No relevant differences in demographic data and comorbidities were found between patients with and without AVWS. Median vWF:Ac/Ag ratio was 0.79 (IQR 0.70–0.89) in the whole study cohort and AVWS (as defined as a vWF:Ac/Ag ratio ≤ 0.7) was present in 36 (25.2%) patients before TAVI. vWF:Ac/Ag ratio was negatively correlated with *P*mean (*r* = − 0.22, *p* < 0.05). At baseline, vWF:Ac/Ag ratio was significantly lower in high gradient AS compared to low gradient AS [0.78 (IQR 0.67–0.86) vs. 0.83 (IQR 0.74–0.93), *p* < 0.05, Fig. 2a] and in BAV compared to TAV [0.70 (IQR 0.63–0.78) vs. 0.81 (IQR 0.71–0.89), *p* < 0.05, Fig. 2b]. AVWS was significantly more prevalent in BAV than in TAV (53.8% vs. 22.3%, *p* < 0.05), while there were no differences in baseline TTE parameters observed between BAV and TAV (SVI, EF, iEAO and *P*mean each *p* = n.s., Table 2).

**Table 1:** Baseline characteristics

	All (*n* = 143)	vWF:Ac/Ag ratio ≤ 0.7 (*n* = 36)	vWF:Ac/Ag ratio > 0.7 (*n* = 107)	*P* value
Age (years)	82 (IQR 78–85)	81 (IQR 75–86)	82 (IQR 78–85)	0.985
Sex, *n* (% male)	79 (55.2%)	18 (50%)	61 (57%)	0.464
BMI (kg/m^2^)	27.2 (IQR 24.1–29.7)	27.1 (IQR 24.2–29.7)	27.2 (IQR 24.0–29.9)	0.963
STS-Score (%)	2.84 (IQR 1.84–4.76)	3.35 (IQR 1.71–4.72)	2.74 (1.87–4.76)	0.759
Hypertension, *n* (%)	130 (90.9%)	33 (91.7%)	97 (90.7%)	0.855
Diabetes, *n* (%)	47 (32.9%)	9 (25%)	38 (35.5%)	0.245
Obesity (BMI ≥ 30 kg/m^2^), *n* (%)	33 (23.1%)	8 (22.2%)	25 (23.4%)	0.888
History of PCI, *n* (%)	32 (22.4%)	8 (22.2%)	24 (22.4%)	0.979
History of CABG, *n* (%)	13 (9.1%)	2 (5.6%)	11 (10.3%)	0.394
Peripheral artery disease, *n* (%)	25 (17.5%)	2 (5.6%)	23 (21.5%)	0.029
COPD, *n* (%)	19 (13.3%)	6 (16.7%)	13 (12.1%)	0.490
History of stroke/ TIA, *n* (%)	21 (14.7%)	6 (16.7%)	15 (14%)	0.698
Permanent dialysis, *n* (%)	3 (2.1%)	1 (2.8%)	2 (1.9%)	0.742
Medication, *n* (%)				
Aspirin	75 (52.4%)	16 (44.4%)	59 (55.1%)	0.266
Clopidogrel	21 (14.7%)	6 (16.7%)	15 (14.0%)	0.698
Ticagrelor	3 (2.1%)	1 (2.8%)	2 (1.9%)	0.742
Prasugrel	0	0	0	–
Phenprocoumon	20 (14%)	8 (22.2%)	12 (11.2%)	0.100
NOAC	21 (14.7%)	4 (11.1%)	17 (15.9%)	0.484
LMWH	7 (4.9%)	3 (8.3%)	4 (3.7%)	0.269
Beta-blocker	93 (65%)	24 (66.7%)	69 (64.5%)	0.812
Digitalis	6 (4.2%)	2 (5.6%)	4 (7.3%)	0.638
Ca-channel blocker	46 (32.2%)	3 (8.3%)	43 (40.2%)	0.000
ACE-I/ARB	105 (73.4%)	24 (66.7%)	81 (75.7%)	0.288
Diuretics	102 (71.3%)	28 (77.8%)	74 (69.2%)	0.323
Aldosterone antagonist	27 (18.9%)	8 (22.2%)	19 (17.8%)	0.554
Aortic valve				
Bicuspid aortic valve, *n* (%)	13 (9.1%)	7 (19.4%)	6 (5.6%)	0.012
Low gradient stenosis, *n* (%)	59 (41.3%)	11 (30.6%)	48 (44.9%)	0.123
Low flow stenosis, *n* (%)	67 (46.9%)	17 (47.2%)	50 (46.7%)	0.959
*P*mean (mmHg)	42 (IQR 33–53)	45 (IQR 34–59)	40 (IQR 32–52)	0.140
SVI (ml/m^2^)	36 (IQR 30–43)	36 (IQR 30–42)	36 (IQR 31–44)	0.632
EF (%)	56 (IQR 51–61)	57 (IQR 51–61)	56 (IQR 51–62)	0.688
iEOA (cm^2^/m^2^)	0.37 (IQR 0.31–0.43)	0.35 (IQR 0.30–0.39)	0.38 (IQR 0.32–0.45)	0.084
vWF parameters				
vWF:Ac (U/dl)	154 (IQR 128–198)	150 (IQR 111–198)	157 (IQR 131–198)	0.351
vWF:Ag (U/dl)	195 (IQR 160–256)	231 (IQR 180–314)	186 (IQR 158–233)	0.002
vWF:Ac/Ag ratio	0.79 (IQR 0.7–0.89)	0.63 (IQR 0.58–0.67)	0.83 (IQR 0.77–0.91)	< 0.001

**Table 2:** Aortic valve morphology and echocardiographic parameters

	All (*n* = 143)	BAV (*n* = 13)	TAV (*n* = 130)	*P* value
*P*mean (mmHg)	42 (IQR 33–53)	41 (IQR 36–54)	42 (IQR 32–53)	0.894
SVI (ml/m^2^)	36 (IQR 30–43)	38 (IQR 30–43)	36 (IQR 30–43)	0.707
EF (%)	56 (IQR 51–61)	55 (IQR 49–60)	57 (IQR 51–62)	0.342
iEOA (cm^2^/m^2^)	0.37 (IQR 0.31–0.43)	0.34 (IQR 0.30–0.43)	0.37 (IQR 0.31–0.44)	0.514
vWF:Ac/Ag ratio	0.79 (IQR 0.70–0.89)	0.70 (IQR 0.63–0.78)	0.81 (IQR 0.71–0.89)	0.019

Echocardiographic parameters and vWF function in bicuspid (BAV) and tricuspid aortic valve (TAV) cohorts

**Fig.3:**
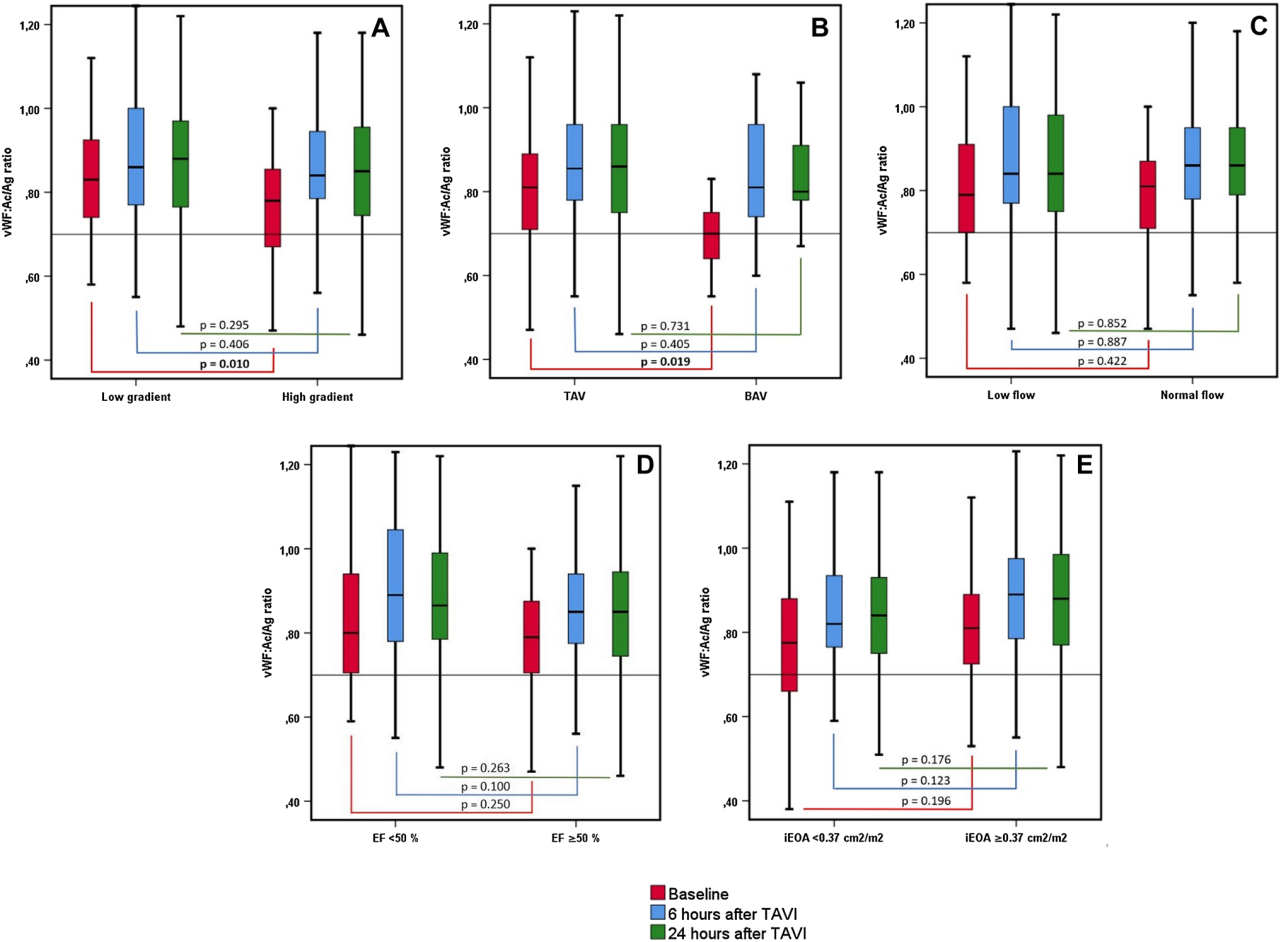
Periprocedural vWF function—different phenotypes of aortic stenosis. Boxplots: vWF:Ac/Ag ratio at baseline, 6 and 24 h after TAVI depending on *P*_mean_ (**A**), valve morphology (**B**), SVI (**C**), EF (**D**) and iEOA (**E**). vWF:Ac/Ag ratio increased significantly from baseline to 6 and 24 h after TAVI in all subgroups (each *p* < 0.05). No significant change from 6 to 24 h after TAVI (each *p *= n.s.).

No differences regarding vWF:Ac/Ag ratio were observed in severe AS classified by SVI (normal flow vs. low flow), EF (preserved EF vs. reduced EF) and iEOA (< vs. ≥ median, Fig. 3).

Before hospital admission, 48 patients (33.6%) were treated with an anticoagulant (Phenprocoumon 14.0%, NOAC 14.7%, LMWH 4.9%) and 82 patients (57.3%) received antiplatelet therapy (Acetylsalicyclic acid 52.4%, Clopidogrel 14,7%, Ticagrelor 2.1%). There was no significant difference in vWF:Ac/Ag ratio between patients with and without anticoagulants or antiplatelet therapy (each *p* = n.s.).

### Bleeding complications

Major bleeding events at least 3 months prior TAVI were recorded in six (4.2%) patients. In two cases the bleeding resulted from vascular complications during coronary angiography. Four (2.8%) patients suffered from TAVI-related life-threatening or major bleeding complication, in all cases associated with major vascular complication according to VARC-2 criteria. A significant difference between patients with or without AVWS was only observed regarding bleeding events occurring 3 months prior TAVI not due to vascular complications (*p* < 0.05, Table 3). These non-vascular major bleeding complications were also significantly more often in patients treated with anticoagulants (*p* < 0.05).

**Table 3:** Periprocedural bleeding events and vascular complications

	All (*n* = 143)	vWF:Ac/Ag ratio ≤ 0.7 (*n* = 36)	vWF:Ac/Ag ratio > 0.7 (*n* = 107)	*P* value
Major bleeding prior TAVI, *n* (%)				
≤ 3 months prior TAVI	6 (4.2%)	3 (8.3%)	3 (2.8%)	0.154
Vascular complication due to PCI	2 (1.4%)	0	2 (1.9%)	0.410
Non-vascular	4 (2.8%)	3 (8.3%)	1 (0.9%)	0.020
Peptic ulcer	3 (2.1%)	2 (5.6%)	1 (0.9%)	0.095
Tooth extraction	1 (0.7%)	1 (2.8%)	0	0.085
TAVI procedure				
Life threatening/ major bleeding, *n* (%)	4 (2.8%)	2 (5.6%)	2 (1.9%)	0.248
Major vascular complication, *n* (%)	5 (3.5%)	2 (5.6%)	3 (2.8%)	0.438

Major bleeding events 3 months prior TAVI, postprocedural bleeding events and vascular complications according to VARC-2 criteria in patients with and without AVWS at baseline

### Postprocedural evolution of vWF:Ac/Ag ratio

**Fig.4:**
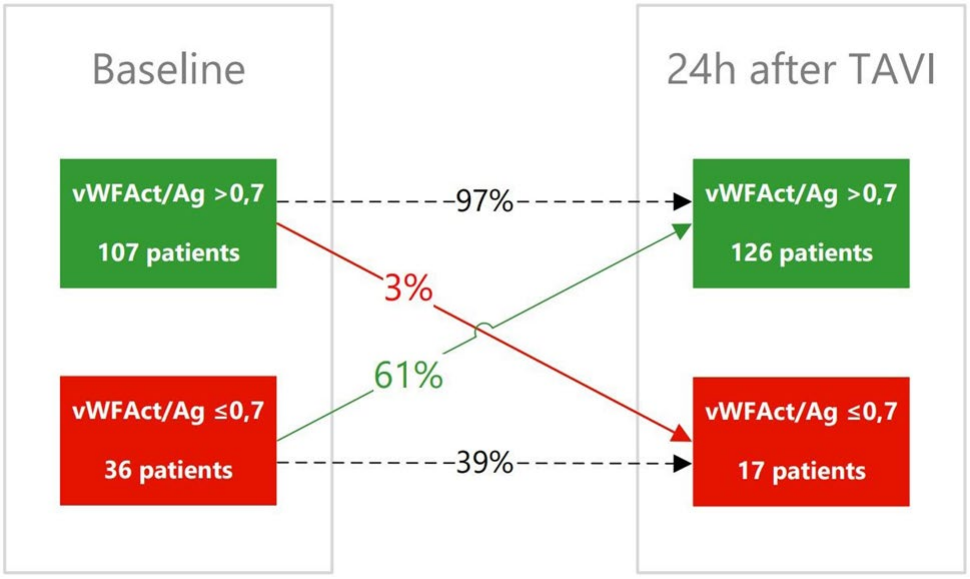
AVWS before and after TAVI procedure. Flowchart: abnormal vWF:Ac/Ag ratio at baseline and 24 h after TAVI. The number of patients with AVWS reduces from 36 (25%) at baseline to 17 (12%) 24 h after TAVI.

In the overall study cohort vWF:Ac/Ag ratio increased significantly from baseline 0.79 (IQR 0.70–0.89) to 0.85 (IQR 0.75–0.96) 24 h after TAVI (*p* < 0.001). In 61% of the patients with AVWS at baseline vWF:Ac/Ag ratio increased to > 0.7 within 24 h after TAVI, while 97% of the patients with a baseline vWF:Ac/Ag ratio ≥ 0.7 remained above this level (Fig. 4). As in the overall study cohort, vWF:Ac/Ag ratio increased significantly in all severe AS subgroups classified by SVI, EF, iEOA, *P*mean and valve morphology within 6 h after TAVI with no further significant increase after 24 h (Fig. 3).

### Device success and vWF function

Pre-discharge TTE showed moderate PVL in 4.2%, where as severe PVL was not detected. Median iEOA after TAVI was 0.91 cm^2^/m^2^ (IQR 0.77–1.09), whereas severe PPM was present in 7.0%. There was no difference in vWF:Ac/Ag ratio 24 h after TAVI between patients with ≥ II° PVL or severe PPM compared to others (each *p* = n.s.). Median mean transprosthetic gradient was 10 mmHg (IQR 7–12) and did not differ significantly between patients with and without AVWS 24 h after TAVI (*p* = n.s.).

### Predictors of AVWS

In the binary logistic regression model including valve morphology, *P*mean, SVI, EF and iEOA, BAV morphology was the only significant independent predictor for vWF:Ac/Ag ratio ≤ 0.7 (*p* < 0.05, all others *p* = n.s.; Table 4).

**Table 4:** Predictors for AVWS—binary logistic regression analysis

	Odds ratio	95% CI	*P* value
BAV	4.06	1.27–13.04	0.018
High gradient	1.55	0.64–3.73	0.328
SVI	1.01	0.97–1.06	0.529
EF	1.01	0.97–1.06	0.552
iEOA	38.09	0.46–3190	0.107

Binary logistic regression analysis with backward elimination regarding vWF:Ac/Ag ratio ≤ 0.7

## Discussion

The aim of the present study was to investigate the prevalence of AVWS in different phenotypes and valve morphologies of severe AS and its course after TAVI. The major findings of this study are that vWF:Ac/Ag ratio did not differ in severe AS subgroups classified by SVI, EF and iEOA, but vWF:Ac/Ag ratio was significantly lower in patients with BAV compared to TAV and in high gradient compared to low-gradient severe AS. Binary logistic regression analysis showed that BAV morphology is a strong predictor for AVWS in severe AS. vWF:Ac/Ag ratio increased in all subgroups and after the procedure there was no significant difference remaining between groups.

### Bicuspid aortic valve morphology leading to AVWS

The current study showed for the first time that valve morphology has an important impact on vWF function in patients with severe aortic stenosis. As previous studies have shown, mean transvalvular gradient was inversely correlated with vWF:Ac/Ag ratio, supporting the hypothesis of high shear stress causing reduced vWF function [6, 7, 9]. The present study demonstrated that patients with BAV had significantly lower levels of vWF:Ac/Ag ratio. According to binary logistic regression analysis, BAV was the only significant predictor for decreased vWF:Ac/Ag ratio. These findings suggest valve morphology to be an additional and independent factor affecting vWF function. Using computational fluid–structure interaction models, Chandra et al. revealed that non-calcified type-1-BAV had a sixfold increase in the temporal shear magnitude (TSM) at the base of the leaflets compared to tricuspid valves [17]. Since similar pressure gradients were applied to all models, increased TSM provides a possible explanation for significantly lower vWF:Ac/Ag ratio in BAV patients.

### Recovery of vWF function after TAVI

King et al. first published in 1987 that recurrent bleeding episodes in patients with aortic stenosis ceased in most cases after surgical aortic valve replacement [18]. Several studies have shown that a restoration of vWF parameters can be obtained with TAVI as well [7, 8, 10, 19]. In our cohort of 143 patients, 25.2% had vWF:Ac/Ag ratio ≤ 0.7 before the intervention. Caspar et al. reported a similar incidence using the same cut-off value [7]. After TAVI the number of patients with AVWS decreased from 25.2 to 11.9% 24 h after the procedure. The significant differences in vWF:Ac/Ag ratio associated with gradient and morphology receded within 6 h after TAVI. This supports the hypothesis that pathological shear stress, which is reduced by valve replacement represents the main reason for AVWS.

Regarding the change in vWF parameters due to valve replacement van Belle et al. have observed an immediate restoration of vWF multimers within 5 min after valve implantation [20]. With a half-life of 6–9 h for vWF antigen [21], reduced degradation cannot be the only reason for the correction of vWF-HMWM within minutes. Therefore, the authors hypothesized that the altered luminal pressure after valve replacement could cause an increased vWF-release from endothelial cells [20].

### Bleeding disorder in patients with severe aortic stenosis

The incidence of AVWS varies widely between studies using different endpoint definitions and laboratory methods ranging between 22.4% [7] and 80% [22]. The interpretation of bleeding complications in the present study is limited by the low amount of bleeding events and by further influencing factors as antithrombotic medication. Patients with AVWS suffered more often from major bleeding not caused by vascular complications, but all four patients were also treated with anticoagulants providing an adequate explanation for an elevated bleeding risk. Furthermore, no patient met all criteria for Heyde’s syndrome, defined as proven bleeding angiodysplasia associated with AVWS. Previous studies using similar endpoint definitions reported an incidence of approximately 3% [19, 23]. All TAVI-related major or life-threatening bleeding events were caused by major vascular complications. Contrary to preprocedural bleeding events not due to vascular injury, TAVI-related major bleeding complications were not associated with AVWS, confirming the finding of previous studies [7, 9].

### Use in clinical practice

Patients with BAV frequently develop symptomatic AS at a younger age compared to patients with TAV [24]. At present patients with BAV are mostly treated with surgical aortic valve replacement (SAVR) [25]. In opposition to TAVI in which most bleeding complications result from vascular complications, SAVR-patients with AVWS are at higher risk for major bleeding [9]. This emphasizes the clinical importance of the finding that BAV is associated with AVWS. However, data comparing the outcome of patients with different valve morphologies after SAVR are missing in the current literature.

More attention for AVWS in patients with aortic stenosis would be desirable not only in patients with general high bleeding risk, but also in younger patients with BAV, potentially representing a high-risk population. Further studies at a larger scale are required to confirm this finding. A higher number of patients with BAV will be necessary to evaluate if relevant differences between different types of BAV exist. Periprocedural management could be adapted to patients with elevated bleeding risk. Some patients could possibly benefit from desmopressin or vWF-concentrate administration before SAVR [26, 27] to reduce life-threatening bleeding complications.

### Limitations

The comparability between studies investigating AVWS is possible only to a limited extent because of various laboratory methods and endpoint definitions being used for AVWS, Heyde’s syndrome, bleeding complications and vWF function. In the present study HMWM-analysis was not conducted. Due to the cohort size and the spread width of vWF parameters the conducted analyses have large confident intervals limiting the transferability of these findings on an individual basis. The incidence for Heyde’s syndrome was lower than that in other studies reported. Possible reasons for this discrepancy are small cohort sizes, different clinical practice concerning the indication for endoscopic examination and the fact that the treatment of high-risk patients became possible only in the last few years with the development of TAVI. Because of the low amount of bleeding complications in the present study it is not possible to distinguish the effect of antithrombotic medication from a possible influence of vWF function on the bleeding risk. Larger cohorts will be required to evaluate the influence of different types of BAV morphology on vWF function.

## Data Availability

The datasets generated and analyzed during the current study are available in the figshare repository, https://figshare.com/articles/dataset/vWF_TAVI_data_xlsx/19609515

## Abbreviations

AS: Aortic stenosis
AVWS: Acquired von Willebrand syndrome
BAV: Bicuspid aortic valve
EF: Ejection fraction
HMWM: High-molecular-weight von Willebrand multimers
iEOA: Indexed effective orifice area
PVL: Paravalvular leak
SVI: Stroke volume index
TAV: Tricuspid aortic valve
TAVI: Transcatheter aortic valve implantation
TTE: Transthoracic echocardiography

## References

[1] van Belle E, Vincent F, Rauch A, Casari C, Jeanpierre E, Loobuyck V, Rosa M, Delhaye C, Spillemaeker H, Paris C, Debry N, Verdier B, Vincentelli A, Dupont A, Lenting PJ, Susen S (2019). von Willebrand factor and management of heart valve disease: JACC review topic of the week. J Am Coll Cardiol.

[2] Leebeek FWG, Eikenboom JCJ (2016). Von Willebrand's disease. N Engl J Med.

[3] Heyde EC (1958). Gastrointestinal bleeding in aortic stenosis. N Engl J Med.

[4] Loscalzo J (2012). From clinical observation to mechanism–Heyde's syndrome. N Engl J Med.

[5] Horiuchi H, Doman T, Kokame K, Saiki Y, Matsumoto M (2019). Acquired von Willebrand syndrome associated with cardiovascular diseases. J Atheroscler Thromb.

[6] Vincentelli A, Susen S, Le Tourneau T, Six I, Fabre O, Juthier F, Bauters A, Decoene C, Goudemand J, Prat A, Jude B (2003). Acquired von Willebrand syndrome in aortic stenosis. N Engl J Med.

[7] Caspar T, Jesel L, Desprez D, Grunebaum L, Samet H, Trinh A, Petit-Eisenmann H, Kindo M, Ohlmann P, Morel O (2015). Effects of transcutaneous aortic valve implantation on aortic valve disease-related hemostatic disorders involving von Willebrand factor. Can J Cardiol.

[8] Spangenberg T, Budde U, Schewel D, Frerker C, Thielsen T, Kuck K-H, Schäfer U (2015). Treatment of acquired von Willebrand syndrome in aortic stenosis with transcatheter aortic valve replacement. JACC Cardiovasc Interv.

[9] Grodecki K, Zbroński K, Przybyszewska-Kazulak E, Olasińska-Wiśniewska A, Wilimski R, Rymuza B, Scisło P, Czub P, Koper D, Kochman J, Pawlak K, Ciepiela O, Grygier M, Jemielity M, Lesiak M, Filipiak KJ, Opolski G, Huczek Z (2019). Pre-procedural abnormal function of von Willebrand Factor is predictive of bleeding after surgical but not transcatheter aortic valve replacement. J Thromb Thrombolysis.

[10] van Belle E, Rauch A, Vincent F, Robin E, Kibler M, Labreuche J, Jeanpierre E, Levade M, Hurt C, Rousse N, Dally J-B, Debry N, Dallongeville J, Vincentelli A, Delhaye C, Auffray J-L, Juthier F, Schurtz G, Lemesle G, Caspar T, Morel O, Dumonteil N, Duhamel A, Paris C, Dupont-Prado A, Legendre P, Mouquet F, Marchant B, Hermoire S, Corseaux D, Moussa K, Manchuelle A, Bauchart J-J, Loobuyck V, Caron C, Zawadzki C, Leroy F, Bodart J-C, Staels B, Goudemand J, Lenting PJ, Susen S (2016). Von Willebrand factor multimers during transcatheter aortic-valve replacement. N Engl J Med.

[11] Baumgartner H, Falk V, Bax JJ, de Bonis M, Hamm C, Holm PJ, Iung B, Lancellotti P, Lansac E, Rodriguez Muñoz D, Rosenhek R, Sjögren J, Tornos Mas P, Vahanian A, Walther T, Wendler O, Windecker S, Zamorano JL (2017). 2017 ESC/EACTS Guidelines for the management of valvular heart disease. Eur Heart J.

[12] Baumgartner H, Hung J, Bermejo J, Chambers JB, Edvardsen T, Goldstein S, Lancellotti P, LeFevre M, Miller F, Otto CM (2017). Recommendations on the echocardiographic assessment of aortic valve stenosis: a focused update from the European Association of Cardiovascular Imaging and the American Society of Echocardiography. Eur Heart J Cardiovasc Imaging.

[13] Yoon S-H, Kim W-K, Dhoble A, Milhorini Pio S, Babaliaros V, Jilaihawi H, Pilgrim T, de Backer O, Bleiziffer S, Vincent F, Shmidt T, Butter C, Kamioka N, Eschenbach L, Renker M, Asami M, Lazkani M, Fujita B, Birs A, Barbanti M, Pershad A, Landes U, Oldemeyer B, Kitamura M, Oakley L, Ochiai T, Chakravarty T, Nakamura M, Ruile P, Deuschl F, Berman D, Modine T, Ensminger S, Kornowski R, Lange R, McCabe JM, Williams MR, Whisenant B, Delgado V, Windecker S, van Belle E, Sondergaard L, Chevalier B, Mack M, Bax JJ, Leon MB, Makkar RR (2020). Bicuspid aortic valve morphology and outcomes after transcatheter aortic valve replacement. J Am Coll Cardiol.

[14] Kappetein AP, Head SJ, Généreux P, Piazza N, van Mieghem NM, Blackstone EH, Brott TG, Cohen DJ, Cutlip DE, van Es G-A, Hahn RT, Kirtane AJ, Krucoff MW, Kodali S, Mack MJ, Mehran R, Rodés-Cabau J, Vranckx P, Webb JG, Windecker S, Serruys PW, Leon MB (2012). Updated standardized endpoint definitions for transcatheter aortic valve implantation: the Valve Academic Research Consortium-2 consensus document (VARC-2). Eur J Cardiothorac Surg.

[15] Zoghbi WA, Asch FM, Bruce C, Gillam LD, Grayburn PA, Hahn RT, Inglessis I, Islam AM, Lerakis S, Little SH, Siegel RJ, Skubas N, Slesnick TC, Stewart WJ, Thavendiranathan P, Weissman NJ, Yasukochi S, Zimmerman KG (2019). Guidelines for the evaluation of valvular regurgitation after percutaneous valve repair or replacement: a report from the American Society of Echocardiography developed in collaboration with the society for cardiovascular angiography and interventions, Japanese Society of Echocardiography, and Society for Cardiovascular Magnetic Resonance. J Am Soc Echocardiogr.

[16] James PD, Connell NT, Ameer B, Di Paola J, Eikenboom J, Giraud N, Haberichter S, Jacobs-Pratt V, Konkle B, McLintock C, McRae S, Montgomery R, R, O'Donnell JS, Scappe N, Sidonio R, Flood VH, Husainat N, Kalot MA, Mustafa RA,  (2021). ASH ISTH NHF WFH 2021 guidelines on the diagnosis of von Willebrand disease. Blood Adv.

[17] Chandra S, Rajamannan NM, Sucosky P (2012). Computational assessment of bicuspid aortic valve wall-shear stress: implications for calcific aortic valve disease. Biomech Model Mechanobiol.

[18] King RM, Pluth JR, Giuliani ER (1987). The association of unexplained gastrointestinal bleeding with calcific aortic stenosis. Ann Thorac Surg.

[19] Sedaghat A, Kulka H, Sinning J-M, Falkenberg N, Driesen J, Preisler B, Hammerstingl C, Nickenig G, Pötzsch B, Oldenburg J, Hertfelder H-J, Werner N (2017). Transcatheter aortic valve implantation leads to a restoration of von Willebrand factor (VWF) abnormalities in patients with severe aortic stenosis - incidence and relevance of clinical and subclinical VWF dysfunction in patients undergoing transfemoral TAVI. Thromb Res.

[20] van Belle E, Rauch A, Vincentelli A, Jeanpierre E, Legendre P, Juthier F, Hurt C, Banfi C, Rousse N, Godier A, Caron C, Elkalioubie A, Corseaux D, Dupont A, Zawadzki C, Delhaye C, Mouquet F, Schurtz G, Deplanque D, Chinetti G, Staels B, Goudemand J, Jude B, Lenting PJ, Susen S (2015). Von Willebrand factor as a biological sensor of blood flow to monitor percutaneous aortic valve interventions. Circ Res.

[21] Sadler JE, Budde U, Eikenboom JCJ, Favaloro EJ, Hill FGH, Holmberg L, Ingerslev J, Lee CA, Lillicrap D, Mannucci PM, Mazurier C, Meyer D, Nichols WL, Nishino M, Peake IR, Rodeghiero F, Schneppenheim R, Ruggeri ZM, Srivastava A, Montgomery RR, Federici AB (2006). Update on the pathophysiology and classification of von Willebrand disease: a report of the subcommittee on von Willebrand factor. J Thromb Haemost.

[22] Frank RD, Lanzmich R, Haager PK, Budde U (2017). Severe aortic valve stenosis. Clin Appl Thromb Hemost.

[23] Godino C, Lauretta L, Pavon AG, Mangieri A, Viani G, Chieffo A, Galaverna S, Latib A, Montorfano M, Cappelletti A, Maisano F, Alfieri O, Margonato A, Colombo A (2013). Heyde's syndrome incidence and outcome in patients undergoing transcatheter aortic valve implantation. J Am Coll Cardiol.

[24] Roberts WC, Ko JM (2005). Frequency by decades of unicuspid, bicuspid, and tricuspid aortic valves in adults having isolated aortic valve replacement for aortic stenosis, with or without associated aortic regurgitation. Circulation.

[25] Hamdan A, Kornowski R (2020). TAVI in bicuspid aortic valve stenosis. Int J Cardiol.

[26] James AH, Eikenboom J, Federici AB (2016). State of the art: von Willebrand disease. Haemophilia.

[27] Charlebois J, Rivard G-É, St-Louis J (2018). Management of acquired von Willebrand syndrome. Transfus Apher Sci.

